# ***Akkermansia muciniphila-*** **derived extracellular vesicles alleviate colitis-related cognitive impairment via tryptophan metabolic reprogramming of the gut‒brain axis**

**DOI:** 10.1080/19490976.2025.2611546

**Published:** 2026-01-06

**Authors:** Xinyang Chen, Qiqiong Li, Wanyu Zhang, Yushan Xu, Xinke Nie, Xindong Wang, Chunhua Chen, Junhua Xie, Shaoping Nie

**Affiliations:** a State Key Laboratory of Food Science and Resources, Nanchang University, Nanchang, China

**Keywords:** *Akkermansia muciniphila*, extracellular vesicles, colitis, gut–brain axis, 5-HT, cognitive impairment

## Abstract

Ulcerative colitis (UC) is a chronic inflammatory bowel disease with systemic manifestations, including cognitive impairment linked to gut‒brain axis dysregulation. While probiotic therapies show promise, their mechanisms in mitigating neuropsychiatric comorbidities remain unclear. Here, we investigated the therapeutic potential of *Akkermansia muciniphila*-derived extracellular vesicles (AmEVs) in a murine model of dextran sulfate sodium (DSS)-induced colitis and associated cognitive deficits. AmEVs administration significantly alleviated colitis severity, as evidenced by improved weight retention, reduced disease activity index scores, and colon length restoration. Concurrently, AmEVs reversed colitis-driven cognitive impairments, restoring Y-maze and novel object recognition performance to baseline levels. Mechanistically, AmEVs repaired intestinal and blood‒brain barrier integrity by upregulating tight junction proteins, suppressed neuroinflammation via reduced hippocampal pro-inflammatory cytokines, and inhibited microglial/astrocyte activation. Gut microbiota analysis revealed that AmEVs-mediated enrichment of beneficial Bifidobacterium and suppression of pathogenic Bacteroides and Mucispirillum, alongside restored short-chain fatty acid (SCFA) production. Crucially, AmEVs bidirectionally regulated tryptophan metabolism, reducing colonic serotonin (5-HT) overproduction while restoring hippocampal 5-HT levels and 5-HT1A receptor expression. This was accompanied by enhanced synaptic plasticity and BDNF upregulation in the hippocampus. Proteomic and biodistribution studies confirmed AmEVs' delivery of metabolic regulators to hippocampal neurons, including the key protein Amuc_1100**,**directly enhancing 5-HT production *in vitro*. Our findings establish AmEVs as a multifaceted therapeutic agent that concurrently resolves gut inflammation and cognitive deficits via gut–brain axis modulation, offering novel strategies for IBD-related neuropsychiatric comorbidities. Further research is warranted to validate critical vesicular components and optimize clinical translation.

## Introduction

Inflammatory bowel disease (IBD), particularly ulcerative colitis (UC), is a chronic inflammatory disorder of the colon characterized by mucosal damage and systemic immune dysregulation. Beyond its gastrointestinal manifestations, IBD has emerged as a significant risk factor for cognitive dysfunction, with a meta-analysis of clinical data reporting accelerated progression of dementia in affected individuals.^1^^,^^2^ This bidirectional interplay between intestinal inflammation and neurological decline is increasingly attributed to dysregulation of the gut–brain axis – complex network integrating neural, endocrine, and immune signaling pathways.^3^ Central to this axis is the gut microbiota, where disruption of symbiotic microbial communities (dysbiosis) triggers intestinal barrier breakdown, systemic inflammation, and subsequent neuroinflammation.^4^ Restoring microbial homeostasis thus represents a strategic therapeutic avenue for addressing both intestinal pathology and associated cognitive impairments.

Recent advances highlight bacterial extracellular vesicles (bEVs) as pivotal mediators of host-microbe crosstalk within the gut–brain axis.^5^ These nanosized lipid bilayers, secreted by commensal and pathogenic bacteria, transport bioactive molecules such as proteins, nucleic acids, and metabolites to distant host tissues.^6^ Probiotic-derived EVs exhibit unique advantages over live bacteria, including enhanced stability, targeted delivery across biological barriers, and avoidance of uncontrolled microbial proliferation.^7^ In the gut, they reinforce epithelial integrity by upregulating tight junction proteins while suppressing NF-κB-driven proinflammatory cascades.^8^^,^^9^ Notably, their capacity to traverse the blood-brain barrier enables direct modulation of neuroimmune responses—evidenced by studies demonstrating EV-mediated inhibition of microglial hyperactivation and restoration of hippocampal serotonin production in depression models.^10^ These properties position bEVs as precision tools for simultaneously targeting gut inflammation and its neurological sequelae.

Among gut symbionts, *Akkermansia muciniphila* stands out for its dual role in mucin layer regeneration and metabolic regulation, constituting 1%–4% of the healthy human gut microbiome.^11^ While *A. muciniphila*-derived EVs (AmEVs) have shown promise in ameliorating colitis through Toll-like receptor 2 (TLR2) signaling suppression and mucosal repair, their potential to mitigate colitis-associated cognitive deficits remains unexplored.^12^^,^^13^ This gap is critical given that cognitive dysfunction in IBD involves interconnected mechanisms—blood‒brain barrier (BBB) leakage, astrocyte reactivity, and tryptophan metabolism disruption—all of which are potentially modifiable by EV-carried neuroprotective cargo.^14^ Here, we demonstrate that AmEVs concurrently resolve intestinal inflammation and reverse cognitive decline by orchestrating gut barrier restoration, tryptophan metabolism recovery, normalization of microglia‒astrocyte, microbiota reconstitution and SCFAs recovery. Our findings establish AmEVs as multifaceted regulators of gut–brain communication, bridging microbial ecology to neuroimmune homeostasis.

## Results

### AmEVs ameliorate DSS-induced colitis through modulation of inflammatory response and mucosal repair

**Figure 1.**
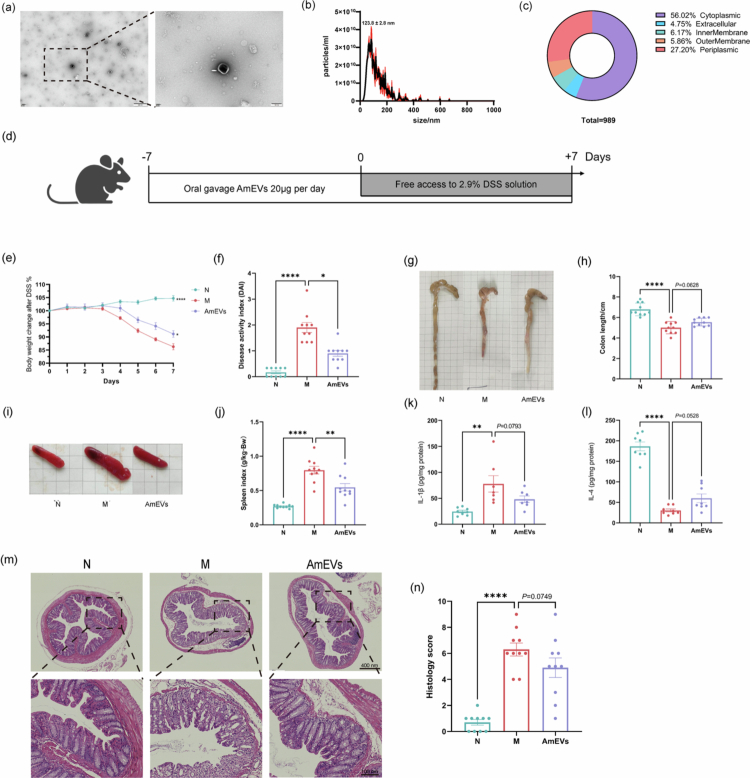
AmEVs alleviate colitis. (a) Transmission electron microscopy characterization of AmEVs, scale bar: 500 nm. (b) Particle size distribution of AmEVs. (c) Proteomic analysis of AmEVs. (d) The design of the animal experiment. (e) Changes in body weight (*n* = 10). (f) Disease activity index scores on the last day (*n* = 10). (g) Representative images of colon length (*n* = 10). (h) Quantification of colon length (*n* = 10). (i) Representative images of the spleen (*n* = 10). (j) Spleen index (*n* = 10). (k and l) Expression of inflammatory factors protein in the colon (*n* = 6‒8). (m) Representative images of H&E staining (*n* = 10), scale bar: 400 μm (upper) and 100 μm (lower). (n) H&E staining score (*n* = 10). Data were shown as means ± SEM. Significance was assessed using the one-way ANOVA test or Kruskal‒Wallis test, giving *p-*values: *p < 0.05, **p < 0.01, and ****p < 0.0001. AmEVs: *Akkermansia muciniphila*-derived extracellular vesicles.

AmEVs were isolated from *A. muciniphila* cultures via ultracentrifugation (Figure S1a). Transmission electron microscopy confirmed their discoid morphology with intact membrane structures (Figure 1a), and nanoparticle tracking analysis shows that their particle size is mainly concentrated at 123.8 nm (Figure 1b). Subcellular localization analysis identified cytoplasmic components as the predominant source of AmEVs, followed by the periplasmic, inner membrane, outer membrane, and extracellular fractions **(**Figure 1c).

In the DSS-induced colitis model (Figure 1d), AmEVs treatment significantly alleviated disease severity. AmEVs-administered mice showed improved body weight retention (Figure 1e) and reduced disease activity index scores (Figure 1f) compared to the model (M) group. Splenomegaly associated with colitis was also mitigated (Figure 1i,j). While colon shortening and histopathological scores exhibited marginal improvement trends (Figure 1g, h, m, and n), these observations suggested enhanced mucosal repair. AmEVs further modulated inflammatory responses in colonic tissues, with trends toward decreased pro-inflammatory IL-1β and increased anti-inflammatory IL-4 levels (Figure 1k,l). Collectively, AmEVs ameliorated colitis progression through the regulation of inflammatory responses and mucosal repair.

### AmEVs alleviate DSS-induced cognitive impairment by modulating neuroinflammation and restoring cytokine balance

**Figure 2.**
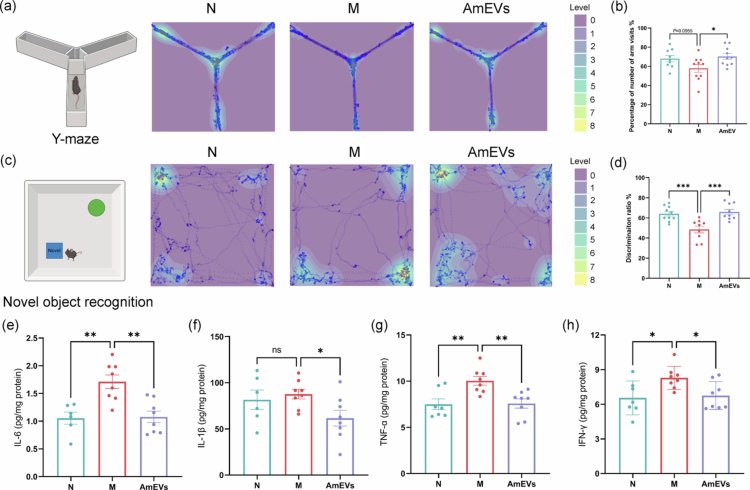
AmEVs alleviate colitis-related cognitive impairment. (a) Representative trajectories heatmap of mice in the Y-maze test. (b) Percentage of the number of arm visits (*n* = 9–10). (c) Representative trajectories heatmap of mice in the NOR test. (d) Discrimination ratio in the NOR test (*n* = 9–10). (e–h) Expression of inflammatory factors protein in the hippocampus (*n* = 6–8). Data were shown as means ± SEM. Significance was assessed using the one-way ANOVA test or Kruskal‒Wallis test, giving *p-*values: *p < 0.05, **p < 0.01, and ***p < 0.001. AmEVs: *Akkermansia muciniphila*-derived extracellular vesicles. NOR: novel object recognition.

DSS-induced colitis triggered spatial and recognition memory deficits. Y-maze spontaneous alternation rates decreased from 68.02% in controls to 57.93% in the colitis mice, with AmEVs treatment restoring performance to 70.32% (Figure 2a,b). Novel object recognition testing demonstrated recovery of discrimination indices from 48.49% to 65.9% following AmEVs administration, reaching levels comparable to the 64.15% observed in healthy controls (Figure 2c,d).

To investigate potential neuroinflammatory mechanisms underlying these cognitive deficits, hippocampal cytokine profiling revealed significant elevation of IL-6, TNF-α, and IFN-γ in colitis mice compared to controls, with IL-1β showing similar upward trends. AmEVs treatment normalized these pro-inflammatory cytokines to baseline levels while reducing IL-1β below control values (Figure 2e–h). Gene expression analysis demonstrated complete restoration of *Il6* mRNA to control levels, whereas *Tnfα* and *Il1β* gene expression remained elevated despite protein-level reductions. Although IFN-γ exhibited discordant transcriptional‒translational dynamics, it ultimately achieved protein concentrations equivalent to those of healthy controls (Figure S2). These results collectively indicate that AmEVs ameliorate colitis-associated cognitive dysfunction through coordinated mitigation of neuroinflammation and restoration of cytokine homeostasis despite persistent transcriptional alterations in specific inflammatory pathways.

### AmEVs reverse colitis-linked dysbiosis via hierarchical microbial remodeling and SCFAs metabolic recovery

**Figure 3.**
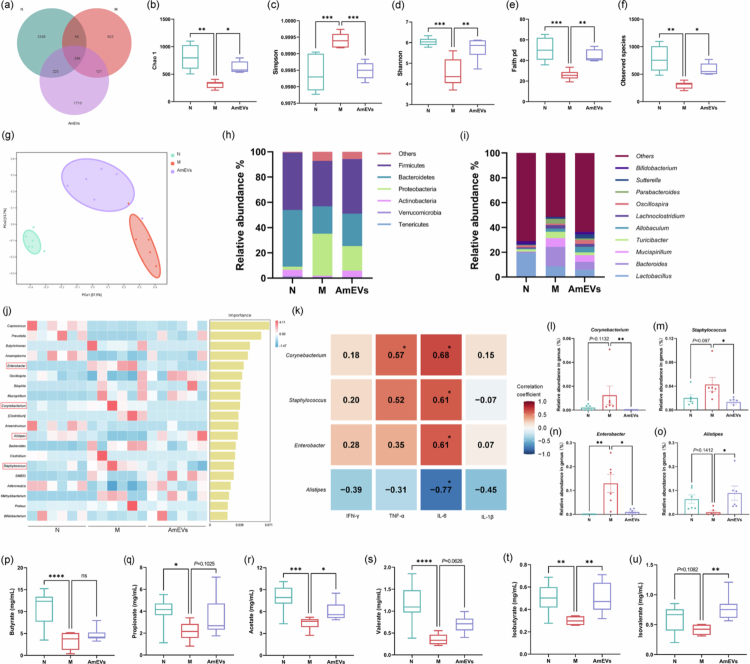
Effects of AmEVs on the gut microbiota and SCFAs in colitis mice. (a) Venn plot. (b–f) *α*-diversity with Chao1(b), Simpson (c), Shannon (d), Faith (e), and observed species (f) (*n* = 6). (g) β-diversity, PCoA plot (*n* = 6). (h) Relative abundance at the phylum level (*n* = 6). (i) Relative abundance at the genus level (*n* = 6). (j) The top 20 discriminative genera by random-forest analysis (*n* = 6). (k) Correlation between differential genera and brain inflammatory cytokines. (l–o) Relative abundance in genus of *Corynebacterium* (l), *Staphylococcus* (m), *Enterobacter* (n), and *Alistipes* (o) (*n* = 6). (p–u) The concentration of butyrate (*p*), propionate (q), acetate (r), valerate (s), isobutyrate (t), and isovalerate (u) in the fecal samples (*n* = 7–8). Data were shown as means ± SEM. Significance was assessed using the one-way ANOVA test or Kruskal‒Wallis test, giving *p-*values: *p < 0.05, **p < 0.01, ***p < 0.001, and ****p < 0.0001. AmEVs: *Akkermansia muciniphila*-derived extracellular vesicles. PCoA: principal coordinate analysis, and SCFAs: short-chain fatty acids.

The gut microbiota serves as a pivotal regulator in the gut–brain axis, with its compositional and functional perturbations constituting hallmark features of inflammatory bowel disease while driving systemic neuroinflammation through metabolite signaling and immune modulation.^15^ 16S rRNA sequencing revealed 5115 amplicon sequence variants (ASVs) across groups, including 2556 unique to the normal (N) group, 603 to the M group, and 1710 to the AmEVs group, with 246 ASVs shared among all groups (Figure 3a). Rarefaction curve stabilization confirmed adequate sequencing depth (Figure S3b–f). Colitis significantly reduced microbial abundance and diversity, which were restored by AmEVs intervention (Figure 3b–f). Principal coordinate analysis revealed distinct clustering patterns, reflecting alterations in the structure of the microbial community induced by DSS and subsequent AmEV treatment (Figure 3g). Phylum-level analysis revealed that both colitis-associated Bacteroidetes depletion and Proteobacteria expansion were reversed by AmEVs administration (Figure 3h). Genus-level profiling identified pathological increases in *Bacteroides* and *Mucispirillum* (positively correlated with colitis^16^^,^^17^) alongside reductions in beneficial *Lactobacillus* and *Bifidobacterium*. AmEVs treatment suppressed colitis-associated genera while enhancing *Bifidobacterium* abundance (Figure 3i). Through random forest analysis, we identified the top 20 potential differential bacterial genera based on their contribution ranks (Figure 3j). To further investigate the potential link between AmEVs-induced gut microbiota remodeling and the alleviation of neuroinflammation, we selected four genera that showed significant differences after AmEVs treatment compared to the M group and performed Spearman correlation analysis with brain inflammatory factors (Figure 3k). The results revealed that *Corynebacterium*, *Staphylococcus*, and *Enterobacter* were positively correlated with multiple pro-inflammatory factors to some extent, which is consistent with previous reports;^18-20^ meanwhile, AmEVs treatment significantly reduced the abundance of these three genera (Figure 3l–n). Notably, *Corynebacterium, Staphylococcus,* and *Enterobacter* showed significant positive correlations with IL-6, while *Staphylococcus* exhibited a positive correlation trend with TNF-α, although not statistically significant (*p* < 0.1). On the other hand, AmEVs treatment significantly reversed the colitis-induced decrease in the abundance of *Alistipes* (Figure 3o). Correlation analysis indicated that *Alistipes* was negatively correlated with all four pro-inflammatory factors, and its correlation with IL-6 was statistically significant, which is also consistent with existing reports.^21^ Additionally, LEfSe analysis (LDA > 2) identified *Allobaculum* as the dominant taxon in AmEVs-treated mice (Figure S1g), a genus implicated in neuroprotection through anti-inflammatory and neurotransmitter modulation.^22^^,^^23^

In addition to the changes in microbial community structure and composition, we also investigated the production of short-chain fatty acids (SCFAs) in mice, which are key microbial metabolites playing an important role in gut health and beyond.^24^ SCFAs analysis demonstrated that colitis-induced depletion of acetic, propionic, butyric, isobutyric, and valeric acids, with isovaleric acid showing a non-significant reduction. AmEVs treatment significantly restored acetic, isobutyric, and isovaleric acid levels, while propionic and butyric acid exhibited recovery trends (Figure 3p–u). Acetic acid restoration is particularly notable given its established role in gut‒brain axis communication and cognitive enhancement.^25^ These findings collectively demonstrate that AmEVs rectify colitis-associated dysbiosis through hierarchical microbial remodeling and SCFA metabolic recovery.

### AmEVs restore gut–brain axis homeostasis via barrier reinforcement, glial deactivation, and synaptic/neuronal repair

**Figure 4.**
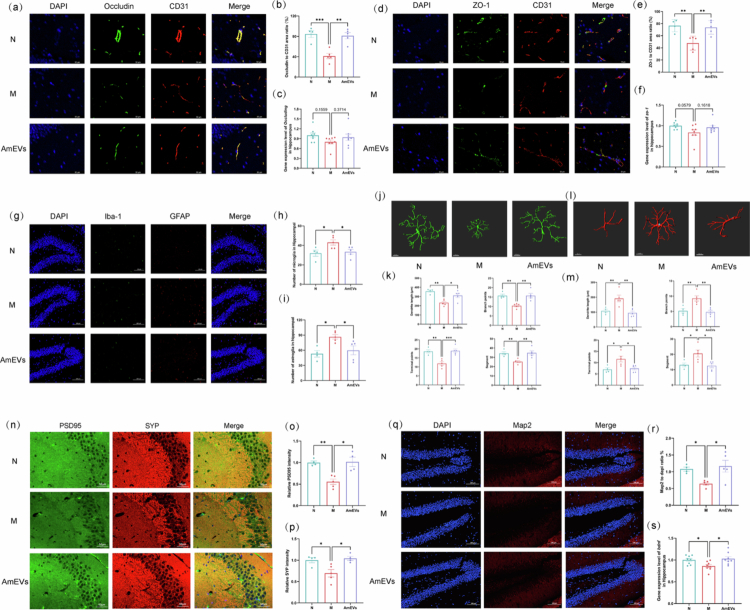
AmEVs restore gut–brain axis homeostasis via barrier reinforcement, glial homeostasis, and synaptic/neuronal repairment. (a) Representative images of Occludin immunostaining in the hippocampus, scale bar: 50 μm. (b) Quantification of Occludin expressed area in the hippocampus (*n* = 4–5). (c) Gene expression levels of *Occludin* in the hippocampus (*n* = 8). (d) Representative images of ZO-1 immunostaining in the hippocampus, scale bar: 50 μm. (e) Quantification of ZO-1 expressed area in the hippocampus (*n* = 4–5). (f) Gene expression levels of *Zo1* in the hippocampus (*n* = 8). (g) Representative co-immunostaining images of Iba-1 and GFAP in the hippocampus, scale bar: 100 μm. (h and i) Quantification of microglia and astrocyte numbers in the hippocampus (*n* = 4–5). (j and k) Representative 3D reconstructions (scale bar: 10 μm) and morphological analysis of microglia (*n* = 4–5). (l and m) Representative 3D reconstructions (scale bar: 10 μm) and morphological analysis of astrocytes (*n* = 4–5). (n) Representative co-immunostaining images of PSD95 and SYP in the hippocampus, scale bar: 50 μm. (o and p) Quantification of PSD95 and SYP expressed intensity in the hippocampus (*n* = 4–5). (q) Representative images of Map2 immunostaining in the hippocampus, scale bar: 100 μm. (r) Quantification of Map2 expressed area in the hippocampus (*n* = 4–5). (s) Gene expression levels of *Bdnf* in the hippocampus (*n* = 8). Data were shown as means ± SEM. Significance was assessed using the one-way ANOVA test or Kruskal‒Wallis test, giving *p-*values: *p < 0.05, **p < 0.01, and ***p < 0.001. AmEVs: *Akkermansia muciniphila*-derived extracellular vesicles. PSD95: Postsynaptic density-95. SYP: Synaptophysin. BDNF: Brain-derived neurotrophic factor.

Emerging evidence highlights the gut‒brain axis as a critical pathway linking intestinal barrier dysfunction to neuroinflammation and cognitive impairment. In DSS-induced colitis, gut microbiota dysbiosis and compromised intestinal tight junctions allow bacterial toxins such as lipopolysaccharides to translocate into the systemic circulation, triggering systemic inflammation and BBB disruption.^26-28^ AmEVs treatment effectively mitigated these pathological cascades. Colonic Occludin protein expression, a key intestinal tight junction marker reduced in colitis mice, was restored to control levels by AmEVs intervention (Figure S4a, b). Although RT-qPCR analysis of *Occludin* mRNA showed no statistical significance among the groups, its expression trend mirrored protein-level changes (Figure S4c). At the BBB level, hippocampal Occludin and ZO-1 proteins, which serve as transmembrane and scaffolding components essential for barrier integrity,^29-31^ showed parallel recovery patterns, with mRNA expression trends mirroring protein-level changes (Figure 4a–f).

Neuroinflammatory responses mediated by glial cells are central to neural damage.^32-34^ Colitis-triggered hippocampal microglial proliferation accompanied by characteristic process retraction and reduced branching complexity, as well as astrocytic hypertrophy marked by excessive process elongation. AmEVs administration normalized the glial cell density and reversed these activation phenotypes. Three-dimensional morphological reconstruction demonstrated the restoration of microglial ramified architecture and astrocytic quiescent morphology (Figure 4g–m).

The gut–brain axis disruption further manifested in synaptic and neuronal alterations. The colitis-induced reductions in presynaptic SYP and postsynaptic PSD95 expression, critical markers of synaptic density,^35^ were fully rescued by AmEVs treatment (Figure 4n–p). Neuronal integrity analysis demonstrated concurrent restoration of microtubule-associated protein 2 (Map2) expression and elevated brain-derived neurotrophic factor (BDNF) mRNA levels, suggesting enhanced neurotrophic support (Figure 4q–s). These findings collectively demonstrate that AmEVs orchestrate comprehensive restoration of gut–brain axis homeostasis through barrier reinforcement, glial deactivation, and synaptic/neuronal repair, thereby counteracting colitis-associated neurocognitive impairments.

### AmEVs restores metabolic homeostasis and neurotransmitter balance by modulating tryptophan metabolism and targeting hippocampal neurons

**Figure 5.**
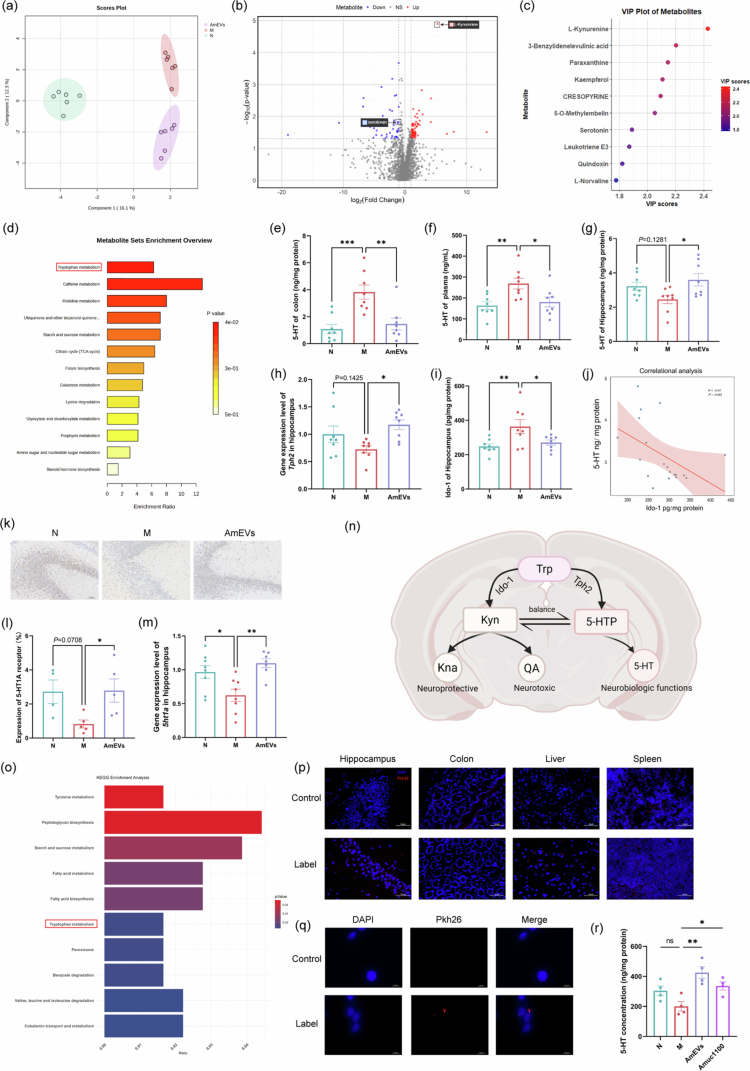
AmEVs bidirectionally regulate tryptophan metabolism in the gut and brain and repair neural damage. (a) Sparse partial least squares discriminant analysis of differential metabolites (*n* = 6). (b) Volcano plot of differential metabolites (*n* = 6). (c) Variable importance in projection analysis for differential metabolites (*n* = 6). (d) KEGG pathway enrichment analysis of differential metabolites. (e–g) Concentration of 5-HT in the colon (e), plasma (f), and hippocampus (g) (*n* = 8). (h) Gene expression levels of *Tph2* in the hippocampus (*n* = 8). (i) The concentration of Ido-1 in the hippocampus (*n* = 8). (j) Correlation analysis of Ido-1 and 5-HT concentrations in the hippocampus. (k) Representative immunostaining images of 5-HT1A receptor in the hippocampus. (l) Quantification of 5-HT1A receptor expressed area in the hippocampus (*n* = 4–5). (m) Gene expression levels of *5ht1ar* in the hippocampus (*n* = 8). (n) Schematic of tryptophan metabolism balance in the brain. (o) KEGG pathway enrichment analysis of AmEVs' the proteomic components. (p) Representative images of the biodistribution of AmEVs in different organs, scale bar: 50 μm. (q) Representative images of HT-22 cell uptake of AmEVs, scale bar: 50 μm. (r) The concentration of 5-HT in HT-22 cells after differentiation. Data were shown as means ± SEM. Significance was assessed using the one-way ANOVA test or Kruskal‒Wallis test, giving *p-*values: *p < 0.05, **p < 0.01, ***p < 0.001, and ****p < 0.0001. AmEVs: *Akkermansia muciniphila*-derived extracellular vesicles.

To determine whether AmEVs affect the metabolism of colitis mice, we conducted untargeted metabolomic analysis on plasma from different groups. Analysis revealed distinct plasma metabolite profiles among the experimental groups, with sparse partial least squares discriminant analysis showing clear separation between healthy controls and colitis mice (Figure 5a). AmEVs treatment substantially modified these metabolic patterns, indicating therapeutic modulation of metabolic reprogramming. Differential analysis using fold change ≥2 and *p* ≤ 0.05 identified 68 upregulated and 42 downregulated metabolites in AmEVs-treated versus untreated colitis mice (Figure 5b), with variable importance in projection values highlighting key contributors (Figure 5c). KEGG pathway analysis identified tryptophan metabolism and primary bile acid production as significantly altered pathways (Figure 5d).

Given the dual relevance of tryptophan metabolism to colitis progression and cognitive function, we focused on serotonin regulation.^36^^,^^37^ Colonic and plasma 5-HT levels increased significantly in the colitis mice versus controls (Figure 5e,f), which AmEVs treatment effectively normalized. Intriguingly, hippocampal 5-HT displayed inverse regulation – colitis induced a downward trend versus N group, which was significantly reversed by AmEVs intervention (Figure 5g). This bidirectional effect paralleled changes in the gene expression of *Tph2*, showing decreased levels in colitis mice and significant upregulation following AmEVs treatment (Figure 5h). Considering the established evidence that microglial activation enhances Ido-1 production—a competing pathway for tryptophan utilization against 5-HT production—we quantified hippocampal Ido-1 levels.^38^^,^^39^ Colitis mice exhibited elevated hippocampal Ido-1 levels versus N group, which were effectively normalized by AmEVs treatment (Figure 5i). Correlation analysis revealed an inverse relationship between hippocampal Ido-1 and 5-HT levels (Figure 5j). Hippocampal 5-HT signaling restoration was further evidenced by AmEVs-mediated recovery of 5-HT1A receptor expression at both the protein and mRNA levels (Figure 5k–m).

Proteomic profiling demonstrated AmEVs enrichment in tryptophan metabolism-associated proteins (Figure 5o). PKH26 tracking confirmed efficient systemic distribution with prominent hippocampal accumulation at 6  h post-administration (Figure 5p). In LPS-challenged differentiated HT-22 neuronal cells, AmEVs not only reversed suppressed 5-HT production but enhanced it beyond baseline levels (Figure 5r), independent of proliferative effects (Figure S5b). Confocal imaging verified the direct neuronal internalization of AmEVs (Figure 5q), supporting their capacity to deliver bioactive components for metabolic regulation. To identify the key protein component within AmEVs responsible for modulating 5-HT production, we focused on Amuc_1100 based on proteomic findings and literature support^40^ (Table S4). Recombinant Amuc_1100 treatment under the same conditions significantly reversed the LPS-induced decline in 5-HT production in differentiated HT-22 cells (Figure 5r). Previous studies suggest that Amuc_1100 modulates tryptophan metabolism via the TLR2 receptor.^40^ Accordingly, co-treatment with the TLR2 inhibitor C29 abolished Amuc_1100-induced 5-HT restoration in LPS-exposed cells (Figure S5g). Interestingly, although C29 partially reduced the promotive effect of AmEVs on 5-HT production, the difference did not reach statistical significance (Figure S5e), suggesting that Amuc_1100 may not be the sole mediator through which AmEVs regulate 5-HT production. Furthermore, inhibition experiments with PCPA (a tryptophan hydroxylase inhibitor) revealed a trend towards suppression of the AmEVs-induced effect, which approached statistical significance (Figure S5f). A similar, though weaker and non-significant, inhibitory trend was observed for Amuc_1100 (Figure S5f).

## Discussion

Our study provides compelling evidence that AmEVs exert multi-dimensional therapeutic effects in colitis and associated cognitive deficits through modulation of the gut‒brain axis. AmEVs administration markedly alleviated colitis severity, as demonstrated by improved body weight retention, reduced DAI scores, and attenuated colon shortening.^41^ These physiological improvements were accompanied with behavioral rescue: AmEVs restored Y-maze and novel object recognition performance to near-baseline levels, underscoring their capacity to counteract colitis-driven cognitive impairment. The concurrent mitigation of gut and brain pathologies highlights the interconnectedness of the gut–brain axis and positions AmEVs as a novel therapeutic agent capable of harmonizing this bidirectional network.

Central to the therapeutic efficacy of AmEVs is their ability to rebalance tryptophan metabolism across the gut–brain axis. In the colon and plasma, AmEVs suppressed excessive 5-HT production, a phenomenon linked to intestinal barrier disruption and autophagy inhibition during colitis.^37^ Conversely, in the hippocampus, AmEVs reversed the colitis-induced decline in 5-HT levels, accompanied by upregulated expression of *Tph2*, the rate-limiting enzyme for 5-HT production. This compartmentalized regulation of 5-HT-suppressing its hyperactivation in the gut while replenishing its neuroprotective pools in the brain-suggests that AmEVs normalize serotonergic signaling in a context-dependent manner. Critically, we identified Amuc_1100—a key protein component of AmEVs—as a principal mediator of this hippocampal 5-HT restoration. Recombinant Amuc_1100 protein alone was sufficient to reverse LPS-impaired 5-HT production in differentiated HT-22 neuronal cultures, mirroring the effects of intact AmEVs. Moreover, this effect was abolished by co-treatment with the TLR2 inhibitor C29, confirming that Amuc_1100 operates, at least partially, through TLR2 signaling. Interestingly, while C29 also partially attenuated the pro-serotonergic effects of whole AmEVs, the persistence of significant rescue suggests that additional TLR2-independent mechanisms or vesicular components may contribute to the full therapeutic response. Critically, hippocampal 5-HT restoration coincided with a marked recovery of 5-HT1A receptor expression, a key mediator of serotonergic neurotransmission implicated in cognitive function.^42^ Previous studies have established that the activation of the 5-HT1A receptor stimulates BDNF production, which is consistent with our observation of elevated *Bdnf* gene levels in colitis mice treated with AmEVs.^43^ BDNF upregulates PSD95, a protein vital for synaptic integrity, to enhance synaptic plasticity and neurodevelopment.^44^ This finding aligns with the observed upregulation of PSD95 and SYP in this study, as well as neuronal repair in hippocampal MAP2 slices. Thus, AmEVs likely alleviate neuronal damage through a sequential cascade involving serotonin availability enhancement, 5-HT1A receptor activation, BDNF-mediated synaptogenesis (as demonstrated by upregulated SYP and PSD95 expression), and ultimately cognitive restoration. Further supporting the centrality of tryptophan metabolic reprogramming, the inhibition of TPH with PCPA showed a strong trend toward blunting the AmEVs-induced increase in neuronal 5-HT production. This pharmacological evidence underscores that AmEVs enhance 5-HT production largely through the canonical TPH-dependent biosynthetic pathway rather than merely inhibiting alternative breakdown routes like the kynurenine pathway.

This bidirectional regulation may be attributed to AmEVs' unique capacity for organ-specific cargo delivery. Crucially, PKH26 tracing demonstrated preferential accumulation of AmEVs in the hippocampus within 6 h post-administration, mirroring the spatial specificity of 5-HT restoration observed *in vivo*. These findings collectively establish that AmEVs act as bacterial-derived nanocarriers capable of (1) traversing the gut‒brain axis, (2) directly engaging with neuronal populations through membrane fusion, and (3) delivering enzymatic machinery to locally rectify serotonin metabolism – a tripartite functionality that synergistically drives the observed neurorepair cascade.

The anti-inflammatory properties of AmEVs further synergize with this neuroprotective pathway. By reducing hippocampal levels of pro-inflammatory cytokines and suppressing microglial/astrocyte activation, AmEVs create a microenvironment conducive to BDNF signaling. Activated glia are known to secrete cytokines such as IL-1β and TNF-α, which suppresses BDNF-induced Akt phosphorylation and exacerbate synaptic loss.^45^ Notably, our 3D morphological analysis revealed that AmEVs could inhibit the transformation of microglia and astrocytes into activated phenotypes, thereby effectively suppressing their pro-inflammatory characteristics. This glial quiescence likely amplifies the benefits of restored 5-HT/BDNF dynamics by removing inhibitory signals that otherwise impede synaptic repair. Concurrently, the repair of intestinal and blood‒brain barriers by AmEVs via the upregulation of Occludin and ZO-1 restricts systemic inflammation and neurotoxic metabolite translocation, further safeguarding hippocampal integrity. For instance, reduced plasma levels of bacterial LPS and pro-inflammatory mediators may prevent BBB leakage, thereby limiting neuroinflammation.^46^ Together, these mechanisms illustrate how AmEVs orchestrate a multi-layered defense against colitis-associated neuropathology.

Our correlation analyses provide further mechanistic links between AmEVs-induced microbial changes and improved brain function. Specifically, we observed that the genera suppressed by AmEVs treatment (*Corynebacterium*, *Staphylococcus*, and *Enterobacter*) were positively correlated with the hippocampal levels of pro-inflammatory cytokines. This suggests that AmEVs may alleviate neuroinflammation, at least in part, by reducing the abundance of these pro-inflammatory taxa. Conversely, AmEVs enriched for *Alistipes*, which showed a negative correlation with IL-6. Importantly, the net effect of AmEVs treatment was a significant reduction in hippocampal cytokine levels, indicating that the overall microbial community shift created by AmEVs favors an anti-inflammatory state in the brain. Additionally, the enrichment of *Bifidobacterium* and suppression of *Bacteroides* and *Mucispirillum* align with prior reports linking these taxa to colitis severity and neuroinflammation.^47^^,^^48^ *Bifidobacterium* species are renowned for their anti-inflammatory properties and ability to enhance gut barrier function through SCFA production,^49^ while *Bacteroides* and *Mucispirillum* are associated with mucosal degradation and immune dysregulation.^50-52^ The increased abundance of SCFAs—particularly acetate—in AmEV-treated mice further supports this gut–brain axis. While SCFAs reinforce intestinal tight junctions to maintain gut barrier integrity, they can also traverse the BBB to suppress neuroinflammation and promote cerebrovascular barrier function.^53^^,^^54^ Notably, these microbiota-related improvements are not isolated events but are functionally interconnected with other observed effects of AmEVs. The restoration of microbial homeostasis and enhancement of gut barrier function likely contribute to reducing systemic inflammation, which subsequently alleviates the burden on the blood‒brain barrier. This creates a more favorable environment for the direct neuroprotective actions of AmEVs, including their regulation of tryptophan metabolism and enhancement of serotonergic signaling in the hippocampus. The coordinated actions of AmEVs on the gut microbiota, barrier function, and neural metabolism collectively form an integrated therapeutic framework that simultaneously addresses both peripheral inflammation and central nervous system dysfunction, ultimately leading to the amelioration of both the intestinal and neurological manifestations of colitis.

**Figure 6.**
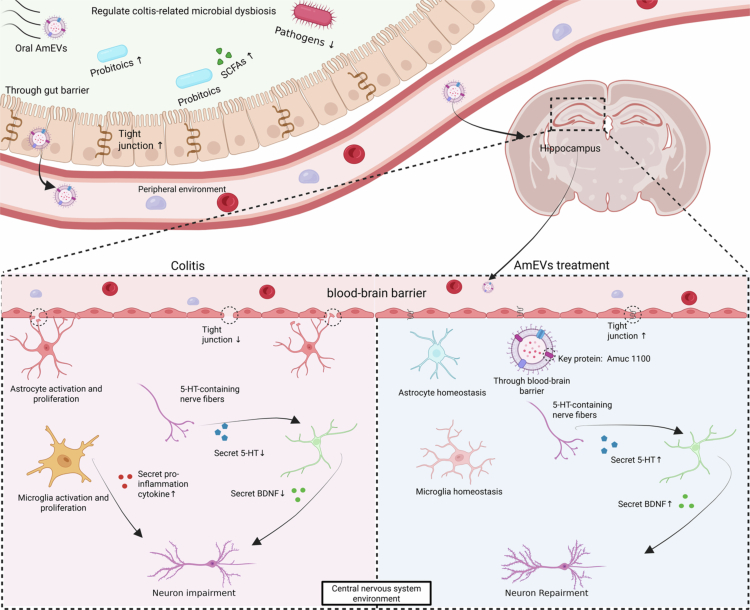
Proposed mechanisms of orally administered AmEVs in treating colitis and associated cognitive impairment via the gut‒brain axis. AmEVs attenuate colitis by reinforcing intestinal barrier integrity and modulating microbial dysbiosis. Following systemic distribution and hippocampal enrichment, AmEVs directly enhance neuronal tryptophan metabolism, restoring physiological 5-HT production and serotonergic signaling. This central action is associated with reduced neuroinflammation (decreased astrocyte/microglia activation), neuronal repair, and BDNF-mediated synaptogenesis. Bidirectional gut‒brain axis modulation collectively restores tissue homeostasis. Created with BioRender.

In conclusion, AmEVs could be a multifaceted therapeutic agent capable of concurrently resolving colitis and its cognitive impairment through spatiotemporal modulation of the gut‒brain axis. Our experimental data suggest that orally delivered AmEVs demonstrate multi-organ biodistribution with apparent hippocampal enrichment, which may contribute to the restoration of physiological 5-HT levels and the upregulation of synaptic plasticity markers in colitis models. This spatial pattern coincided with mitigated pathological 5-HT hyperactivation in the gastrointestinal system; however, the mechanistic connections between the gut and brain effects remain to be fully elucidated. This bidirectional efficacy is mediated by three synergistic mechanisms: peripheral reinforcement of intestinal barrier integrity, central delivery of bacterial-derived metabolic enzymes to hippocampal neurons, as evidenced by direct 5-HT production enhancement in neuronal cultures, and systemic suppression of neuroinflammatory cascades. The observed recovery of serotonergic neurotransmission, BDNF-mediated synaptogenesis, and MAP2-positive neuronal repair collectively validate AmEVs' capacity to orchestrate cross-tissue homeostasis (Figure 6). These findings redefine probiotic-derived EVs as natural nanotherapeutics with inherent brain-targeting specificity, offering a novel strategy to combat IBD-associated neuropsychiatric comorbidities.

## Limitations of the study

Although Amuc_1100 emerged as a key protein within AmEVs capable of enhancing hippocampal 5-HT production via TLR2 signaling, its contribution relative to other vesicular constituents—such as additional proteins, lipids, or nucleic acids—remains unquantified. The fact that TLR2 inhibition only partially abolished AmEVs' effects further suggests the involvement of parallel or compensatory mechanisms. Combinatorial inhibition strategies or CRISPR-based functional screens will be essential to map the full repertoire of bioactive elements driving the therapeutic response.

Our functional validation, while informative, relied primarily on pharmacological inhibitors in cellular models. These findings now require genetic confirmation *in vivo*, ideally through neuron-specific knockout models targeting *Tph2* or *Tlr2*, to establish the necessity of these pathways in mediating the cognitive benefits of AmEVs.

The correlations observed between specific bacterial genera and neuroinflammatory markers, though mechanistically suggestive, do not establish causality. Whether microbiota shifts are necessary or sufficient for the neuroprotective effects of AmEVs remains an open question. Fecal microbiota transplantation from AmEV-treated donors, or targeted microbial interventions, will be critical to test this hypothesis directly.

Another major gap lies in the potential role of neural routes—particularly the vagus nerve—in mediating AmEVs' central effects. Given its established function as a key conduit for gut-to-brain signaling, subdiaphragmatic vagotomy combined with AmEV administration could help disentangle humoral from neural contributions to the observed outcomes.

## Methods

### Extraction of AmEVs

*A. muciniphila* was obtained from the State Key Laboratory of Food Science and Resources, Nanchang University. The bacterium was cultured anaerobically at 37 °C for 48 h in BHI medium (Hope Bio-technology, China). Following incubation, the culture was centrifuged (12,000 rpm, 20 min). Sequential filtration of the supernatant through 1.2, 0.45, and 0.22 μm filters removed bacterial debris and impurities. The clarified filtrate was then subjected to ultracentrifugation (Beckman Coulter, Fullerton, CA, USA) at 150,000 × *g* and 4 °C for 2 h to pellet the microspheres. The resulting pellet was resuspended in sterile phosphate-buffered saline (Figure S1). AmEVs suspended in PBS were quantified for protein concentration using a BCA assay kit (Beyotime Bio-technology, China). Finally, the aliquoted samples were stored at −80 °C.

### Transmission electron microscopy analysis

Transmission electron microscopy (TEM) was employed to examine the morphology of AmEVs. Briefly, a 20 μL aliquot of the AmEVs solution was applied to a copper grid. After 1 min of air-drying, surplus liquid was carefully removed using filter paper. The grid was then negatively stained by applying an equal volume (20 μL) of 2% phosphotungstic acid (SPI-Chem, USA) and allowing it to stand for 3 min. Excess stain was subsequently blotted away with filter paper. Following complete drying, the samples were visualized using a JEOL JEM1400 TEM (Japan).

### NanoSight analysis

The detection cell was washed with deionized water, and the instrument was calibrated using polystyrene microspheres (100 nm). The detection cell was then re-washed with PBS buffer solution. The samples were diluted 20-fold and 40-fold with PBS buffer solution, respectively, and then subjected to detection, with each sample measured three times. The results were processed, and the size and concentration of the exosomes were analyzed using the NanoSight NS300 system (NanoSight, UK).

### Biodistribution of AmEVs

PKH26-labeled AmEVs were administered to C57BL/6J mice. After 6 h, the mice were sacrificed, and their brains, colons, livers, and spleens were immediately embedded using an embedding agent for frozen sections. Cryostat sections of the aforementioned tissues were prepared, and after DAPI staining, an anti-fluorescence quenching agent was applied for mounting. Images were captured using a confocal laser scanning microscope.

### Animal models

The animal experiments were conducted in accordance with the Guidelines for the Care and Use of Laboratory Animals set out by the National Institutes of Health. They were also approved by the Experimental Animal Care and Use Committee of Nanchang University, with approval number being NCULAE-20250529001. The experimental mice were 6-week-old male C57BL/6J mice, which were divided into three groups: the normal group (N), the model group (M), and the AmEVs-treated group (AmEVs). There were 15 animals in each group, with 10 used for body weight, behavioral, and subsequent molecular experiments, and the remaining 5 were perfused with paraformaldehyde via the heart at sacrifice for immunofluorescence and immunohistochemical experiments of brain tissue. The experiment lasted for 21 d, during which all mice were allowed to acclimate in the animal room for one week after arrival. After the acclimation period, each mouse in the AmEVs group was administered with 200 μL of AmEVs suspension (containing 20 μg of AmEVs protein) via gavage, while the M group mice were given the same volume of PBS by gavage. On day 15, the drinking water of the M and AmEVs group mice was replaced with a 2.9% DSS solution. Behavioral tests were conducted on days 20 and 21. On day 22, after the completion of the breeding period, all the mice were sacrificed and subjected to cardiac perfusion. Subsequently, blood, colonic contents, cecal contents, spleen, and brain tissue were collected for further analysis.

### Behavioral test

The Y-maze and novel object recognition tests were employed to evaluate the mice's spatial working memory and short-term recognition memory. In the Y-maze test, the mice were placed in a maze with three equal-sized arms at 120° angles to each other. A camera recorded their movements as they explored for 5 min. Entries into each arm and the time spent there were recorded to calculate the alternation accuracy. For the novel object recognition test, the mice explored two identical objects in a white square open box. After a 10-min interval, one object was replaced, and the mice exploration times were recorded to calculate the discrimination index. To ensure data validity, we applied a pre-defined inclusion criterion of ≥5 s total object exploration time during the test session; animals failing to meet this threshold were excluded from analysis, as insufficient exploration prevents meaningful interpretation of cognitive preference. All apparatuses were cleaned with 20% ethanol between trials to eliminate odor cues. Behavioral trajectories were analyzed using ImageJ and visualized using R studio.

### Histopathology analysis

Colon tissues underwent fixation in 4% paraformaldehyde, followed by paraffin embedding and sectioning at 4 μm. Sections were stained with hematoxylin and eosin (H&E). Histopathological scoring of H&E-stained sections was performed based on established criteria (Supplementary Table 2), with the total score representing the sum of all individual indices. For immunohistochemistry (IHC), antigen retrieval was achieved via microwave treatment. After natural cooling, the sections were washed on a decolorizing shaker. Subsequent immunostaining involved incubation with a primary antibody against 5-HT1A (Servicebio, China; 1:500 dilution), followed by a horseradish peroxidase (HRP)-conjugated secondary antibody (Goat Anti-Mouse IgG, Servicebio, China; 1:200 dilution), and visualization using DAB substrate. Finally, the nuclei were counterstained with hematoxylin, and the sections were dehydrated and mounted.

### Immunofluorescence staining

Paraffin-embedded tissue sections underwent dewaxing and hydration. Antigen retrieval was performed via high-pressure heat treatment, followed by natural cooling and washing on a decolorizing shaker. Immunostaining utilized primary antibodies targeting ZO-1, Occludin, and Iba-1 (1:500; Abcam, UK), along with anti-GFAP and Map2 (1:500; Servicebio, China). Subsequently, sections were incubated with species-specific secondary antibodies: Alexa Fluor 488-conjugated goat anti-mouse IgG, Alexa Fluor 568-conjugated goat anti-rat IgG, and Alexa Fluor 633-conjugated goat anti-rabbit IgG (all at 1:500; Thermo Fisher Scientific, USA). After secondary antibody incubation, the nuclei were counterstained with DAPI. Confocal imaging was conducted using a Leica Stellaris STED super-resolution microscope (Germany).

### Extraction and real-time quantitative PCR analysis of mouse hippocampal and colon RNA

Total RNA was extracted from hippocampal and colonic tissues using TRIzol. Reverse transcription was performed using the Takara cDNA Reverse Transcription Kit, followed by qPCR amplification with the corresponding reagents. The primer sequences used for RT-qPCR are listed in Supplementary Table 3. Relative gene expression levels were determined using the 2^−ΔΔCt^ method.

### Enzyme-linked immunosorbent assay (ELISA) and cytokines microarray assay

Total hippocampal and distal colon tissues were homogenized in PBS. The tissue homogenate was centrifuged at 3000 rpm for 20 min, and the supernatant was stored at −80 °C. For the plasma, blood was collected and processed into plasma by centrifugation and then stored at −80 °C. The levels of 5-HT in the colon, hippocampus, and plasma were measured using ELISA kits (Elabscience, China). The levels of Ido-1 in the hippocampus and inflammatory cytokines in the colon were also measured using ELISA kits (Elabscience, China; Cuasbio, and China). Specifically, owing to the limited sample size of the hippocampus, the levels of inflammatory cytokines in the hippocampus were assessed using the Luminex Bio-Plex system. Protein levels were normalized to the total protein concentration, which was determined using a BCA kit (Beyotime, China).

### SCFAs quantitative analysis

Gas chromatography was utilized to analyze cecal SCFAs, following previously established methods.^55^ Approximately 100 mg of fecal material were weighed, mixed with 10 volumes of PBS, and supplemented with steel beads. The mixture was homogenized at high speed for 1 min using a tissue grinder (Servicebio, China), followed by centrifugation at 13,000 rpm for 5 min. The supernatant was filtered through a 0.22 μm aqueous filter tip. Subsequently, 0.7 mL of the filtrate was transferred to a 1.5 mL centrifuge tube and mixed with 0.2 mL of 10% sulfuric acid for 1 min. Then, 0.4 mL of anhydrous ether was added, stirred well, and allowed to settle for 2 min before being centrifuged again at 13,000 rpm for 2 min. The resulting supernatant was filtered through a 0.22 μm organic filter tip. SCFAs concentrations were quantified by gas chromatography, with the sample SCFAs content determined via an external standard method.

### Fecal genomic DNA extraction and 16S-rRNA sequencing

Fecal microbial DNA was isolated from mouse feces employing the OMEGA Soil DNA Kit (Omega Bio-Tek, USA). DNA concentration and integrity were assessed using a Nanodrop NC2000 spectrophotometer (Thermo Fisher Scientific, USA) and agarose gel electrophoresis, respectively. PCR amplification targeting the V4 hypervariable region of the bacterial 16S rRNA gene utilized primers 338F (5′-barcode-ACTCCTACGGGAGGCAGCA-3′) and 806 R (5′-GGACTACHVGGGTWTCTAAT-barcode-3′). Following the quantification of individual amplicons, equimolar amounts were pooled. Paired-end sequencing (2 × 250 bp) was executed on an Illumina NovaSeq platform with the NovaSeq 6000 SP Reagent Kit (500 cycles). Microbiome bioinformatics utilized QIIME2 (version 2022.11). The DADA2 plug-in implemented quality control, denoising, paired-read merging, and chimera filtering. The sequences were clustered into amplicon sequence variants (ASVs) at 100% similarity, generating corresponding ASV tables with abundance data. Taxonomy was assigned against the Greengenes reference database (v13.8).

### NoN targeted metabolomics

Plasma metabolite extraction followed modified standard protocols. A mixed solvent of acetonitrile and water (4:1, v/v) was prepared, pre-cooled for 2–3 h, and then used for extraction. Specifically, 200 μL of plasma was combined with an equal volume (200 μL) of this chilled solvent, followed by vortex mixing for 1 min. To maximize the extraction yield, the mixture underwent 20 min of sonication in an ice-water bath. Subsequent centrifugation (16,000 × *g*, 4 °C, 20 min) separated the phases. The resulting supernatant was collected, passed through a 0.22 μm organic membrane filter, and prepared for analysis. Metabolite profiling was conducted using a Shimadzu Nexera X2 UPLC system hyphenated to an AB SCIEX TripleTOF 5600 mass spectrometer. Chromatographic separation employed an ACQUITY UPLC HSS T3 column (2.1 × 100 mm, 1.8 μm) maintained at 40 °C. The raw mass spectral data were processed utilizing Progenesis QI software (v2.0, Waters Corporation). Metabolite annotation involved matching the acquired MS and MS/MS spectra against the HMDB (v4.0) and METLIN (v1.0.6499.51447) databases. Finally, data analysis and visualization were performed using MetaboAnalyst 6.0 (https://www.metaboanalyst.ca) and R Studio.

### DIA proteomics analysis

AmEVs were subjected to DIA proteomics analysis as follows: 300 μL of 8 M urea solution and protease inhibitors (10% of the lysate) were added to the AmEVs sample. After centrifugation at 14,100 × g for 20 min, the supernatant was collected to determine the protein concentration, and the remaining protein was stored at −80° C. Then, 100 μg of protein was taken and reduced with DTT, followed by digestion with trypsin. The sample was desalted using a C18 column. For LC‒MS/MS analysis, a Q Exactive HF-X mass spectrometer coupled to an EASY nLC 1200 system was used. The peptides were separated and detected in DDA mode, and the data were acquired in DIA mode. For data analysis, a hybrid spectral library was generated in Spectronaut software to identify and quantify proteins, and KEGG analysis and protein interaction prediction were also performed.

### Cell culture

HT-22 cells (a mouse hippocampal neuronal cell line) purchased from Shanghai Zhong Qiao Xin Zhou Bio-technology were cultured in DMEM medium with 10% fetal bovine serum (FBS), 100 IU/L penicillin and 10 μg/mL streptomycin at 37 °C with 5% CO₂. For differentiation, HT-22 cells were cultured in differentiation medium (neurobasal medium with 2 mmol/L glutamine and 0.5 × N2 supplement).^56^

After differentiation, the cells were divided into three groups. Group N received regular culture medium. Group M received medium with 1 μg/mL LPS. The AmEVs group received medium with 1 μg/mL LPS plus 10 μg/mL AmEVs. The Amuc_1100 (FineTest, China) group received medium with 1 μg/mL LPS plus 0.5 μg/mL Amuc_1100. All the inhibitors were used at 25 μM, a concentration selected based on cell-viability assays. All groups were co-incubated for 24 h, after which the proteins were collected for ELISA testing.

### Cellular uptake of AmEVs

Consistently with previous experiments, AmEVs were labeled with PKH26 and co-incubated with HT-22 cells that had adhered for 12 h for a period of 6 h. Subsequently, the culture medium was discarded, and the cells were rinsed three times with PBS. Then, DAPI staining was performed. After mounting, the samples were observed under a fluorescence microscope.

## Supplementary Material

Supplementary material Supplementary_material clean.docx

## Data Availability

The sequence data that support the findings of this study have been deposited in the NCBl database (PRJNA1272734; http://www.ncbi.nlm.nih.gov/bioproject/1272734). Details are provided within the manuscript.
