# Supplementary material for: Akkermansia muciniphila- derived extracellular vesicles alleviate colitis-related cognitive impairment via tryptophan metabolic reprogramming of the gut‒brain axis
Source: Gut Microbes. 2026 Jan 6;18(1):2611546. doi: 10.1080/19490976.2025.2611546 (PMC12785224; doi:10.1080/19490976.2025.2611546)
Supplement: Supplementary material — Supplementary_material clean.docx [file KGMI_A_2611546_SM4443.docx]

***Akkermansia muciniphila*-derived extracellular vesicles alleviate colitis-related cognitive impairment via tryptophan metabolic reprogramming of the gut-brain axis**

Xinyang Chen^1^, Qiqiong Li^1^, Wanyu Zhang^1^, Yushan Xu^1^, Xinke Nie^1^, Xindong Wang^1^, Chunhua Chen^1^, Junhua Xie^1,*^ and Shaoping Nie^1,*^

^1^ State Key Laboratory of Food Science and Resources, Nanchang University, Nanchang 330047, China.

* Corresponding Author:

Prof. Junhua Xie ([junhuax@ncu.edu.cn](mailto:junhuax@ncu.edu.cn))

Prof. Shaoping Nie ([spnie@ncu.edu.cn](mailto:spnie@ncu.edu.cn)) Tel & Fax: +86-0791-88304452

**Table S1 Disease Activity Index**

| Progress | Extent | Score |
| --- | --- | --- |
| Fecal morphology | Normal | 0 |
|  | Mild diarrhea | 1-2 |
|  | Mild liquid stool | 3-4 |
| Bleeding | No bleeding | 0 |
|  | Mild bleeding | 1-2 |
|  | Massive bleeding | 3-4 |
| Weight changes | Weight loss ＜ 1% | 0 |
|  | Weight loss 1%-5% | 1 |
|  | Weight loss 5%-10% | 2 |
|  | Weight loss 10%-15% | 3 |
|  | Weight loss ＞ 15% | 4 |

**Table S2 Histopathologic Scoring**

| Progress | Extent | Score |
| --- | --- | --- |
| inflammatory extent | No inflammation | 0 |
|  | Infiltration of inflammatory cells in the base of the crypt | 1 |
|  | Inflammatory cell infiltration into the muscularis mucosae | 2-3 |
|  | Infiltration in the muscularis mucosae, mucosal thickening and edema | 4 |
| Epithelial cell damage | No damage | 0 |
|  | Decrease in epithelial cells | 1 |
|  | Massive reduction in epithelial cells | 2-3 |
|  | Complete loss of epithelial cells | 4 |
| Crypt damage | Normal | 0 |
|  | Partial loss of crypt structure | 1 |
|  | Crypt enlargement or extensive crypt loss | 2-3 |
|  | Complete absence of crypt structure | 4 |

**Table S3 Disease Activity Index**

| Gene | Forward | Reverse |
| --- | --- | --- |
| *Il1β* | CACCTCACAAGCAGAGCACAAG | GCATTAGAAACAGTCCAGCCCATAC |
| *Tnfα* | ACCCTGGTATGAGCCCATATAC | ACACCCATTCCCTTCACAGAG |
| *Il6* | TAGTCCTTCCTACCCCAATTTCC | TTGGTCCTTAGCCACTCCTTC |
| *Occludin* | CCAGGCAGCGTGTTCCT | TTCTAAATAACAGTCACCTGAGGGC |
| *Zo1* | AGGACACCAAAGCATGTGAG | GGCATTCCTGCTGGTTACA |
| *Hprt* | AGTGTTGGATACAGGCCAGAC | CGTGATTCAAATCCCTGAAGT |
| *Tph2* | GCAAGACAGCGGTAGTGTTCT | CAGTCCACGAAGATTTCGACTT |
| *5htr1a* | GACAGGCGGCAACGATACT | CCAAGGAGCCGATGAGATAGTT |
| *Bdnf* | TCATACTTCGGTTGCATGAAGG | ACACCTGGGTAGGCCAAGTT |

Note: The primers for *Ifnγ* are sourced from commercial primers of Beyotime Biotech Inc (QM03298S).

**Table S4 Identification of Amuc_1100 in AmEVs by DIA-MS**

| **Protein** | **Peptide sequence** | **Charge** | **Precursor.Id** |
| --- | --- | --- | --- |
| Amuc_1100 | AINSLVNK | 2 | AINSLVNK2 |


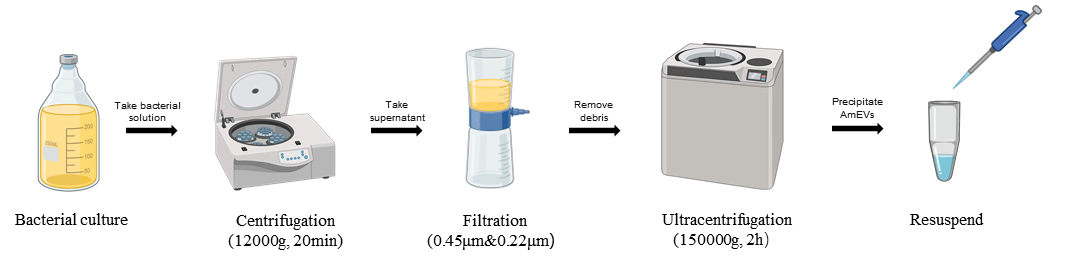


**Fig. S1 The extraction process of *Akkermansia muciniphila*-derived extracellular vesicles.** *Akkermansia muciniphila* was cultured in brain heart infusion broth medium under anaerobic conditions at 37 °C for 48 hours. The culture was then centrifuged at 12,000 rpm for 20 minutes, and the supernatant was filtered through 1.2 μm, 0.45 μm, and 0.22 μm filters to remove debris. The filtrate was ultracentrifuged at 150,000g and 4 °C for 2 hours, and the resulting pellet was resuspended in sterile phosphate-buffered saline.


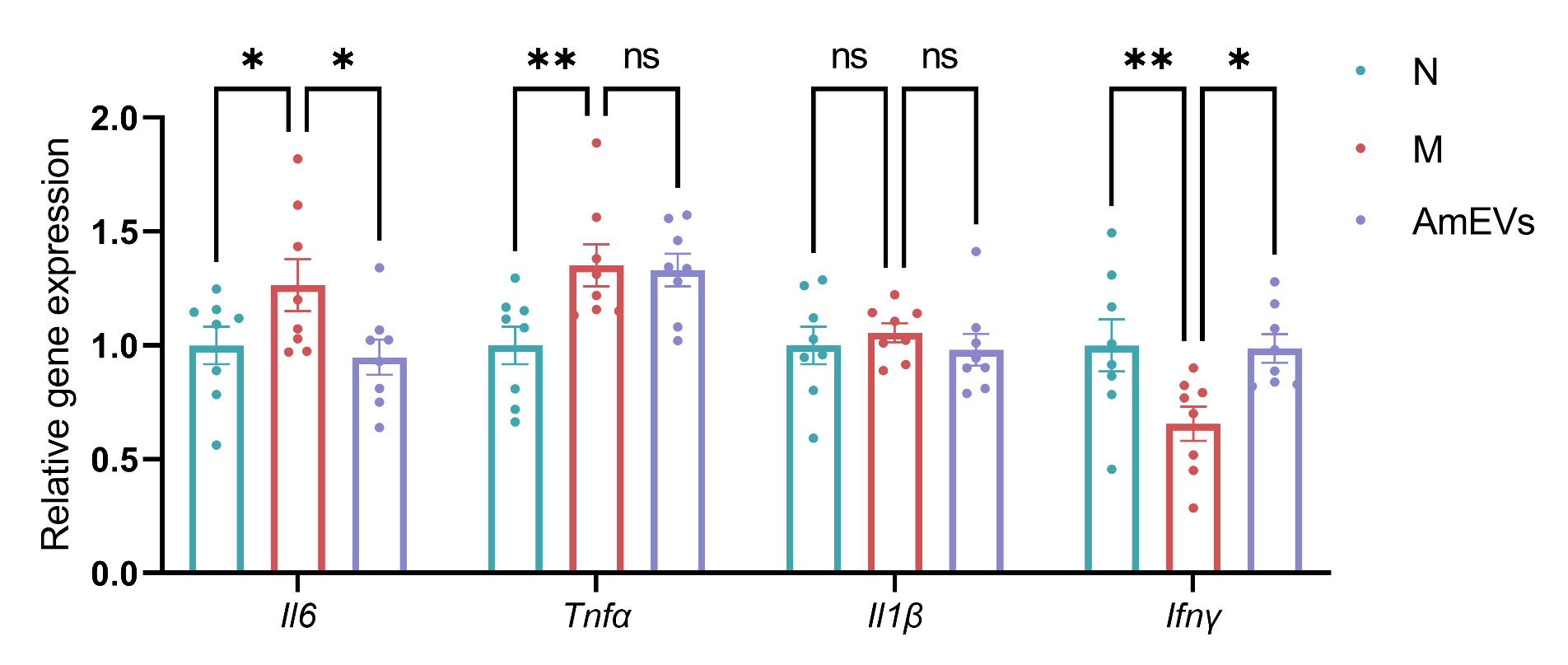


**Fig. S2 Expression of inflammatory factors gene in hippocampus.** Data were shown as means ± SEM (n = 7-8). Significance was assessed using the one-way ANOVA test or Kruskal-Wallis test, giving P values: **P* < 0.05, and ***P* < 0.01. AmEVs: *Akkermansia muciniphila*-derived extracellular vesicles.


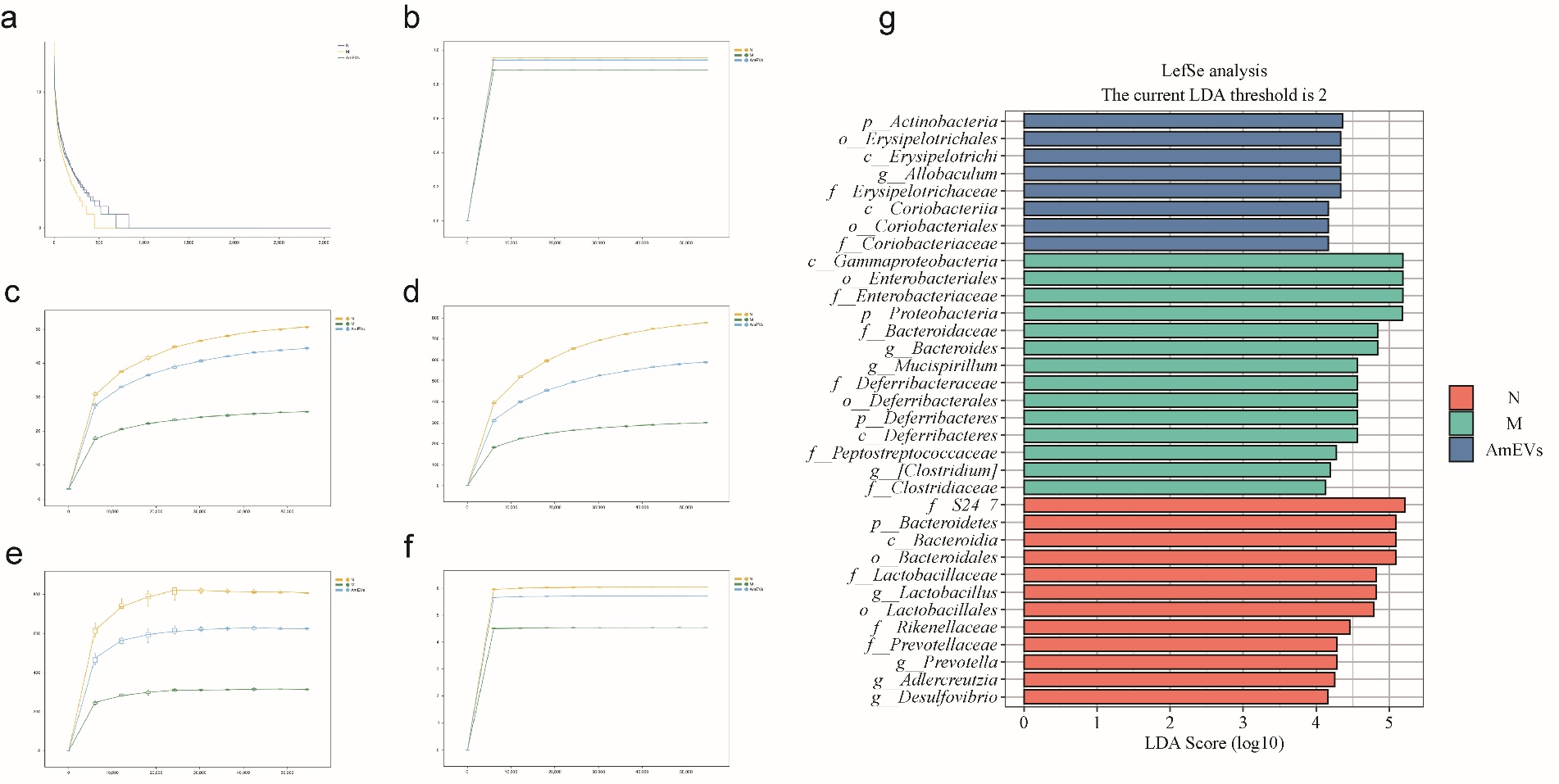


**Fig. S3 Effects of AmEVs on gut microbiota in colitis mice. （a）**Abundance curve. **(b-f)** Sparse curve of Chao1 (b), Faith (c), Observe (d), Shannon (e) and Simpson index (f). **(g)** LEfSe analysis LDA bar chart.


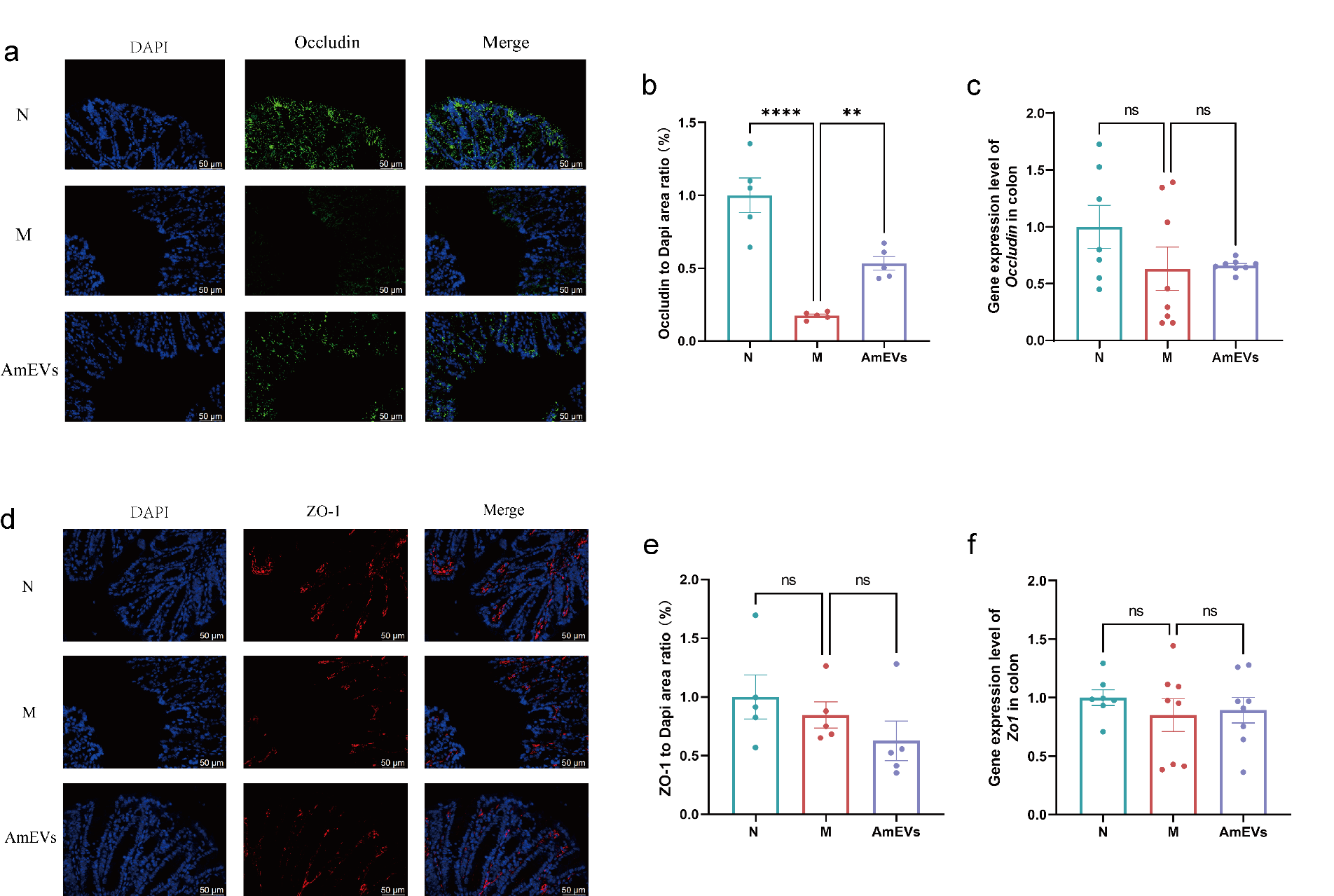


**Fig. S4 AmEVs repair the gut barriers. (a)** Representative images of Occludin staining in the colon. **(b)** Quantification of Occludin expressed area in colon (n = 4-5). **(c)** Gene expression levels of *Occludin* in the colon (n = 4-5). **(d)** Representative images of ZO-1 staining in the colon. **(f)** Gene expression levels of *Zo1* in the colon (n = 8). Data were shown as means ± SEM. Significance was assessed using the one-way ANOVA test or Kruskal-Wallis test, giving P values: ***P* < 0.01, and *****P* < 0.0001. AmEVs: *Akkermansia muciniphila*-derived extracellular vesicles.


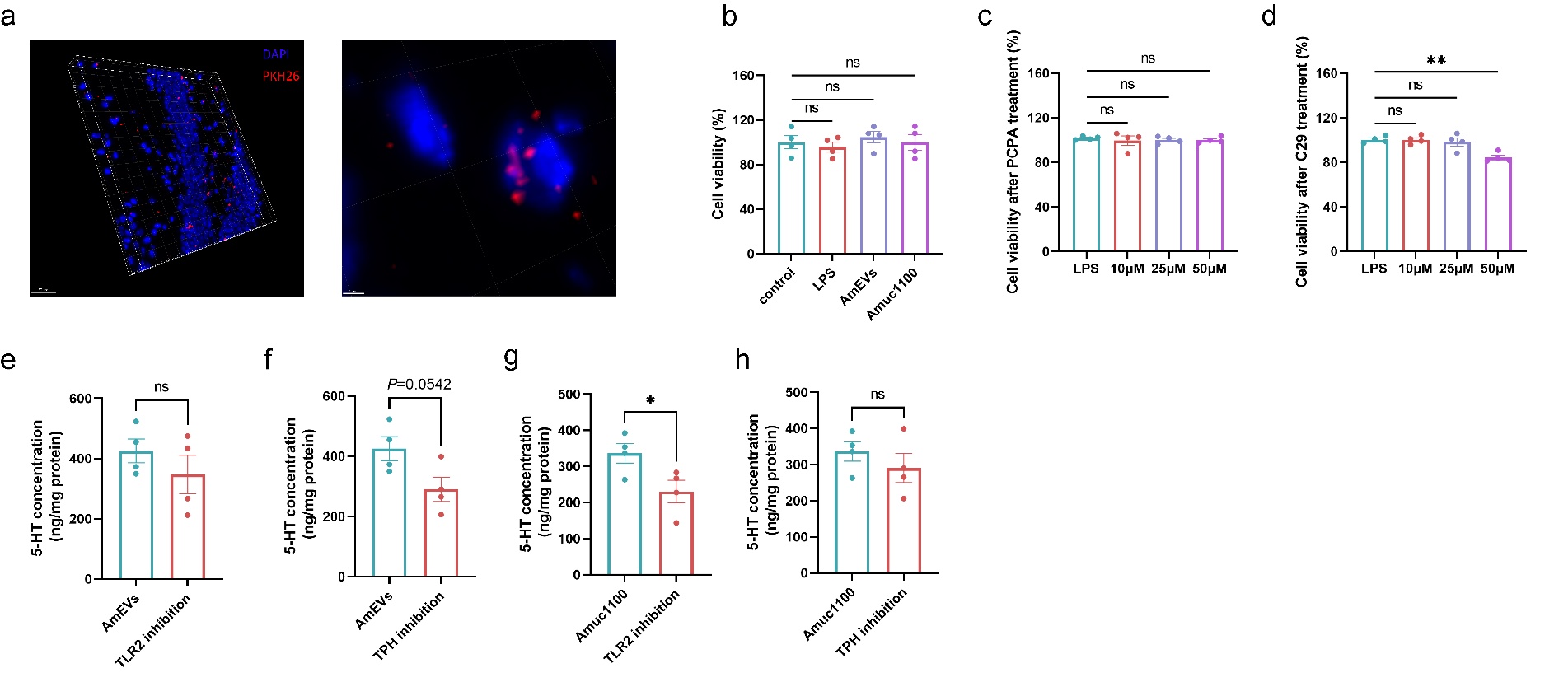
 **Fig. S5 The delivery capacity of AmEVs and their impact on the proliferation of HT-22 cells. (a)** AmEVs enrichment in mouse hippocampal tissue, scale bar: 50 μm and 2 μm. **(b-d)** Cell viability assessed by CCK-8 assay. Data were shown as means ± SEM (n = 4). **(e-f)** Intracellular 5-HT levels in the AmEVs group after TLR2 (e) and TPH (f) inhibition. **(g-h)** Intracellular 5-HT levels in the Amuc1100 group after TLR2 (g) and TPH (h) inhibition. Significance was assessed using the one-way ANOVA test, Kruskal-Wallis test, and T-test, giving P values: **P* < 0.05, ***P* < 0.01. AmEVs: *Akkermansia muciniphila*-derived extracellular vesicles.
